# Non-invasive testing to diagnose the underlying auditory pathology of hearing impairment

**DOI:** 10.1016/j.heares.2025.109391

**Published:** 2025-08-10

**Authors:** Neil J Ingham, Karen P Steel

**Affiliations:** Wolfson Sensory, Pain and Regeneration Centre, King’s College London, London SE1 1UL, UK

**Keywords:** Mutant mice, Cochlear pathology, Auditory Brainstem Response, Distortion Product Otoacoustic Emission, Diagnostic Testing, Site-of-lesion

## Abstract

Of the three major classifications of inner ear pathology, sensory, neural and metabolic, it is relatively easy to distinguish a sensory pathology from a neural or inner hair cell pathology using current clinical tools. Deficiencies in both distortion product otoacoustic emissions (DPOAE) and auditory brainstem responses (ABR) suggest a sensory pathology involving outer hair cells. Normal DPOAEs with deficiencies in ABRs suggest a neural pathology, involving inner hair cells, auditory nerve synapses or neural deficiencies. However, there are currently no non-invasive methods to distinguish a metabolic pathology from sensory or neural pathologies that may be suitable for translation into a clinical setting. Here, mutant mouse lines with defined sites-of-lesion in the inner ear are used to evaluate a method published by Mills (2006) to differentiate these main classes of inner ear pathology. ABR and DPOAE thresholds were recorded from mutant mice and littermate controls. Plotting normalised DPOAE thresholds against ABR thresholds resulted in a continuum of responses from normal to severe threshold elevations for both the ABR and DPOAE recordings. These responses did not fall neatly in the different pathology zones suggested by Mills, or into elliptical clusters suggested by the recordings made in exemplar mutant lines in the current study. Examination of extended parameters, such as response amplitude, did not provide any further differentiation between mice carrying different pathologies. We conclude that measurement of ABR and DPOAE thresholds and response amplitudes does not provide sufficient information to stratify metabolic inner ear pathology from either sensory or neural pathologies.

## Glossary

ABR: Auditory Brainstem Response
DPOAE: Distortion Product Otoacoustic Emissions
EP: Endocochlear Potential
f1: Lower frequency tone used for DPOAE measurements
f2: Upper frequency tone used for DPOAE measurements
IHC: Inner Hair Cell
OHC: Outer Hair Cell
SD: Standard Deviation

## 1 Introduction

In recent years there has been increasing interest in the development of treatments for hearing impairment, including gene therapy to target genetically-diagnosed deafness in children (Lv et al., 2024; Qi et al. 2024; Wang et al. 2024). However, the majority of people affected by hearing loss have no genetic diagnosis and it is likely that more generic approaches to treatment based on the varied sites-of-lesion within the inner ear will be developed. The development of such treatments requires better diagnostic tools than we currently have available to stratify types of hearing impairment for clinical trials, provide more sensitive outcome measures, and decide which treatment is appropriate for each affected person. For example, there would be no point in attempting to trigger hair cell regeneration if there is a primary stria vascularis defect and no endocochlear potential (EP). There is an unmet clinical need to differentiate between the different inner ear pathologies and non-invasive electrophysiological recordings may fill this gap in clinical diagnostic testing.

There are many forms of inner ear sensorineural hearing loss, with many causes, but based on human temporal bone pathology, most cases fall into three major categories (Schuknecht and Gacek 1993); (1) Sensory (including outer hair cell dysfunction), (2) Metabolic (including stria vascularis dysfunction), and (3) Neural (auditory neuron or inner hair cell defects).

It is already possible to distinguish between neural (abnormal IHC or neural function) and sensory hearing loss (dysfunction of IHCs and OHCs) by comparison of Auditory Brainstem Response evoked potentials and Distortion Product Otoacoustic Emissions (DPOAEs). A neural pathology will have normal DPOAEs (as OHCs are unaffected) whilst ABRs are impaired (due to IHC / Neural dysfunction). A sensory pathology will have abnormal DPOAEs and ABRs. In animals, it is possible to assess strial dysfunction by measuring the endocochlear potential (EP), the high positive potential of the scala media compartment within the cochlea, but this is a terminal procedure so is unsuitable for translation to a clinical tool.

**Fig. 3:**
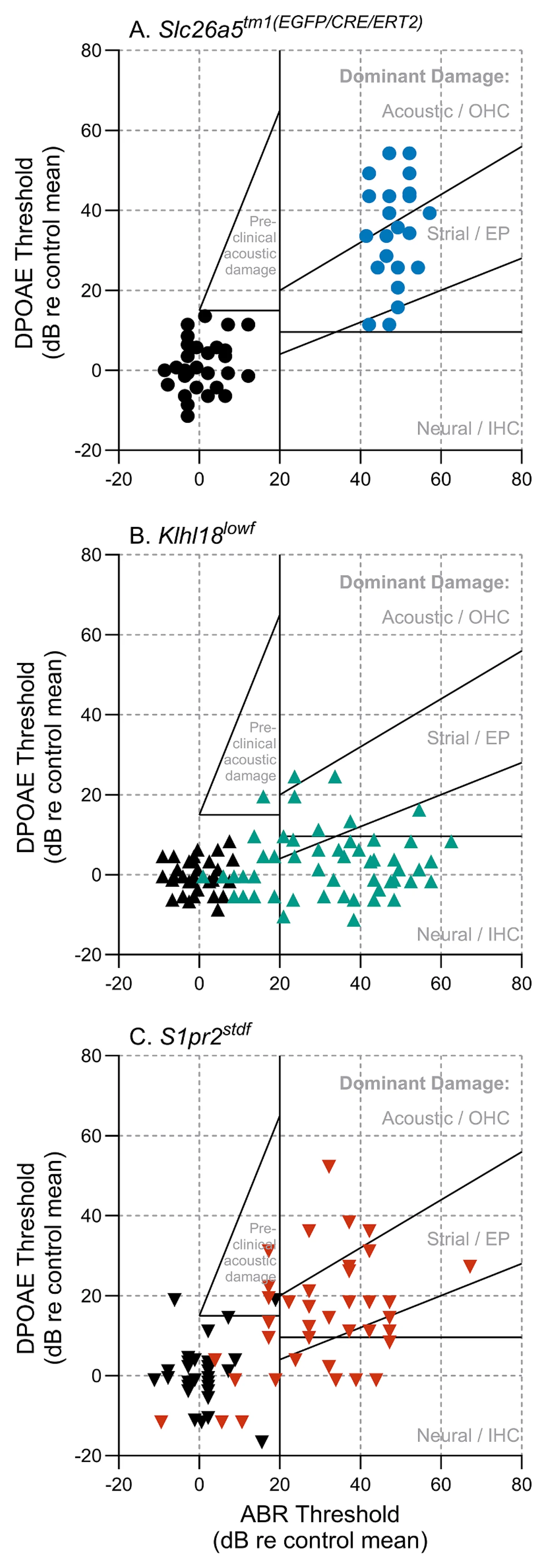
Distributions of normalised thresholds. Normalised ABR & DPOAE thresholds from (A) *Slc26a5^tm1^* control (black circles) and mutant mice (blue circles), (B) *Klhl18^lowf^* control (black up-triangles) and mutant mice (green up-triangles) and (C) *S1pr2^stdf^* control (black down-triangle) and mutant mice (red down-triangles), are plotted to overlay the zones for different pathologies defined by Mills (2006), shown in grey. Each point represents responses at one frequency from one mouse.

There is no obvious way of distinguishing a primary stria vascularis defect from a primary hair cell or neural defect in humans, but these are very different types of defect that will require different treatments. To date, the best approach suggested in humans is an analysis of the shape of the pure-tone audiogram (eg Schuknecht 1994; Vaden et al. 2022), where the different types of hearing impairment outlined by Schukneckt & Gacek (1993) are reflected in different patterns when audiometric thresholds are plotted. However, many audiogram shapes often do not fall into clearcut categories (Mills 2006; Vaden et al. 2022) so there is a need for additional diagnostic information. A strial deficiency resulting in a reduced EP affects both ABR and DPOAE measurements by reducing the electrochemical gradient driving signal transduction in both IHCs and OHCs; ABR and DPOAE response amplitudes are reduced and thresholds become elevated under these circumstances. One scheme proposed to diagnose the pathology of hearing impairment was proposed by Mills (2006; see also Fig. 3) based on data from gerbils (Mills and Schmiedt 2004). Gerbils that had been noise-exposed (to produce OHC damage), chronically exposed to furosemide (to induce a reduced EP), or untreated control animals had both ABR and DPOAE measurements recorded. The hypothesis was that a reduced EP will affect transduction of both IHCs and OHCs due to a reduced driving force across transduction channels, but the responses of IHCs additionally will be affected by the reduced amplification of the organ of Corti provided by the activity of OHCs, thus receiving a double hit from a reduction in EP (Mills 2006). Therefore, the normalised ABR and DPOAE thresholds from each frequency stimulation of each gerbil were plotted against each other and the slopes fell into different zones. Responses from noise-exposed animals showed approximately linear relationships between DPOAE and ABR thresholds as predicted. In contrast, responses from furosemide-treated animals showed approximately twice as much ABR threshold change compared with DPOAE threshold change, again as predicted by the hypothesis. From this scheme, a third zone was proposed where animals with a neural hearing defect would be predicted to fall, corresponding to elevations of ABR threshold with normal DPOAE thresholds.

In the study presented here, we use a number of mutant mouse lines where hearing pathology has been previously characterised, including exemplars of the three main hearing impairment pathologies. We investigate whether these hearing-impaired mutant mice exhibit patterns of ABR and DPOAE responses that allow for a differential diagnosis of the inner ear pathology following the scheme described by Mills (2006).

## 2 Materials and methods

### 2.1 Ethics statement

Mouse studies were carried out in accordance with UK Home Office regulations and the UK Animals (Scientific Procedures) Act of 1986 (ASPA) under UK Home Office licences, and the study was approved by the King’s College London Animal Welfare and Ethical Review Body. Mice were culled using methods approved under these licences to minimize any possibility of suffering.

### 2.2 Mutant mouse lines

Mutant mice used in this study were generated at the Wellcome Sanger Institute on a C57BL/6N genetic background (Ingham et al. 2021; Lewis et al. 2022; White et al., 2013) and their allele details and pathologies are summarised in Table 1. Three mutant mouse lines were used as exemplars of the main types of inner ear pathology: *Slc26a5^tm1^* with an OHC defect to represent “sensory” pathology, *Klhl18^lowf^* with an IHC defect to represent “neural” pathology and *S1pr2^stdf^* to represent “metabolic” pathology. *Slc26a5* encodes prestin, a protein mostly expressed in OHCs, and the mutant mice used have raised thresholds for both DPOAEs and ABRs across all frequencies tested with little change with increasing age (Lachgar-Ruiz et al. 2024). *Klhl18^lowf^* mutants in contrast have normal DPOAEs suggesting normal OHC function, and at early stages they have normal ABR thresholds but from around 4 weeks old ABR thresholds increase, particularly at low frequencies. IHC stereocilia of *Klhl18^lowf^* mutants show abnormal tapering at their tips especially in the apical part of the cochlea corresponding to low frequency detection but there was no sign of reduced synapse numbers under IHCs suggesting a primary IHC defect (Ingham et al. 2021). The earliest defect found in *S1pr2^stdf^* mutants is a reduced endocochlear potential associated with raised ABR thresholds at 4 weeks old followed by progressive increase in thresholds until no responses were detected at older ages (Ingham et al. 2016).

A further 6 mutant lines were analysed as other pathological examples. Oncomodulin is primarily expressed in OHCs and the *Ocm^tm1e^* mutants have normal ABR thresholds at 4 weeks old followed by progressive loss of hearing affecting high frequencies first (Lachgar-Ruiz et al. 2024).*Klc2^tm1e^* mutants have progressive increase in ABR thresholds starting at low frequencies at two weeks old but progressing to all frequencies later, accompanied by loss of OHCs, evidence of reduced OHC synapses and reduced labelling of Kcnma1 (BK channels) in IHCs at 4 weeks old (Ingham et al. 2019). The *Mir182* knockouts exhibited a mild hearing loss starting at higher frequencies and progressing with age to lower frequencies with no obvious structural correlates, but as this microRNA is expressed in both IHCs and OHCs it is likely that both are involved in the pathophysiology (Lewis et al., 2020). *Pex3^tm1a^* homozygotes showed progressive increase in ABR thresholds at high frequencies from 3 weeks onwards, with reduced synapse counts and raised DPOAE thresholds by 4 weeks old (Kochaj et al. 2022). *Pcdh15^em2Wtsi^* mutant mice with a D435A mutation affecting the calcium binding site of Pcdh15 showed progressive high frequency hearing loss from 4 weeks onwards. As Pcdh15 is a component of the tip link, both IHCs and OHCs are expected to be involved in the pathophysiology. Finally, *Wbp2^tm2a^* mutants were studied, which show swollen nerve endings below IHCs and increased DPOAE thresholds, implicating both IHCs and OHCs in their pathology (Buniello et al., 2016).

These mice were all tested at the youngest age where hearing-impairment is evident (Table 1) to maximise the chances that they experience only their primary pathophysiology at this age, rather than an older age where secondary pathologies may become involved.

Younger mice carrying mutations in *S1pr2* and *Klhl18* before their hearing loss pathologies emerge were also studied, and mice with a second, targeted, mutation of *Klhl18* (*Klhl18^tm1a^*, see Ingham et al. 2021), where responses were recorded from a single cohort of mice across increasing age from 3 weeks old (before emergence of the hearing loss) to 14 weeks old (8 weeks after emergence of the hearing loss).

### 2.3 Anaesthesia

All recordings were performed in animals anaesthetised with either urethane (0.1 ml / 10 g bodyweight of a 20 % w/v solution of urethane in water) or a mixture of 100 mg/kg ketamine hydrochloride (Ketamidor, Chanelle Pharma) and 10 mg/kg xylazine (Rompun, Bayer Animal Health) given by intraperitonteal injection. All mice were subject to measurement of both ABRs and DPOAEs across a range of frequencies, so that response parameters obtained from both techniques could be compared within individual mice. If mice were measured on multiple occasions, their recovery from Ketamine/Xylazine anaesthesia was promoted by atipamezole hydrochloride, 1mg/kg, intraperitoneal (Antisedan, Zoetis).

### 2.4 Auditory brainstem response (ABR) measurements

Brainstem auditory evoked potentials were measured using the method described in detail by Ingham et al. (2011) and Ingham (2019). In brief, anaesthetised mice were placed on a heated blanket in a sound attenuating chamber and positioned to face a loudspeaker (Tucker Davis Technologies TDT, FF1 magnetic speaker). Under control of custom software, calibrated acoustic stimuli were generated, amplified and presented to the mouse via a TDT RZ6 multifunction processor, which also gathered digitised evoked potential recordings from subcutaneous needle electrodes via a TDT RA4LI / RA4PA low impedance headstage / preamplifier. Tone pip stimuli (5 ms duration, with a 1 ms onset and offset ramp) of frequencies ranging from 6 – 36 kHz (in 6 kHz intervals) were presented to the mouse at levels ranging from 0–95 dB SPL (in 5 dB steps) at a rate of 42.3 stimuli per second. Averaged evoked potential responses (256 sweeps per trace), 20 ms in duration, were recorded and saved for offline analyses.

### 2.5 Distortion product otoacoustic emission (DPOAE) measurements

We made measurements of the 2f1-f2 DPOAE component in mice using the method described by Ingham et al. 2021. In brief, stimuli were generated and DPOAE responses recorded using a TDT RZ6 multifunction processor, under the control of TDT BioSigRZ software. Calibrated f1 and f2 frequency tones were presented to the left ear of each mouse, via either TDT EC1 electrostatic speakers or TDT MF1 magnetic speakers coupled to an Etymotic ER10B+ microphone assembly which in turn was placed close to the opening of the ear canal. Frequencies for f2 matched the ABR tone-pip frequencies used (6 - 36 kHz, in 6 kHz intervals). The f1 and f2 frequencies were presented in a ratio of 1: 1.2, with the f2 component presented at −10 dB to 65 dB SPL and f1 presented 10 dB higher than f2. Online Fast Fourier Transformation of the ER10B+ microphone signal by BioSigRZ generated power spectra for each stimulus configuration, from which various features of interest (f1 and f2 stimulus components, the main 2f1-f2 DPOAE, and the measurement noise-floor) were obtained. At the completion of the procedure, mice were culled using cervical dislocation whilst still anaesthetised or recovered for subsequent recording at a later age.

### 2.6 Data analyses

In each mouse for each measurement type and for each stimulus frequency tested, response parameters were extracted for comparison. If there was no response at the maximum stimulus output (95 dB SPL for ABRs, 75 dB SPL for DPOAEs), no datapoint was plotted for that condition.

*ABR thresholds.* From the ABR recordings, response threshold (dB SPL) was determined by visual inspection of a stacked plot of response traces as the minimum dB SPL level where a response was visible and consistent with responses at higher stimulus levels (Ingham et al. 2011).

*DPOAE thresholds*. For DPOAE recordings, 2f1-f2 threshold (dB SPL) was determined as the lowest f2 stimulus level where the distortion product amplitude became consistently higher than 2 standard deviations (SD) above the recording noise-floor (Ingham et al. 2011).

*Normalised thresholds*. Normalised ABR thresholds were calculated as the difference between each response threshold and the mean threshold of control mice at that stimulus frequency. Normalised DPOAE thresholds were calculated as the difference between each response threshold and the mean threshold of control mice at that f2 stimulus frequency. Normalised threshold values <0 were responses where threshold was below the control mean threshold; values >0 were responses where thresholds were higher than the control mean threshold.

*Response amplitudes*. Normalised ABR amplitudes were calculated as the ABR wave 1 amplitude (peak-to-peak) of the response evoked at maximum stimulus intensity (95 dB SPL) for each frequency divided by the mean ABR wave 1 amplitude for control mice at that stimulus frequency. Normalised ABR amplitude values <1 were responses were where the amplitude was below the control mean amplitude; values >1 were responses where the amplitude was higher than the control mean amplitude. Normalised DPOAE amplitudes were calculated at the 2f1-f2 amplitude (dB SPL) at maximum stimulus level (75 dB SPL) subtracted from the mean 2f1-f2 amplitude at maximum stimulus level from responses from control mice at each stimulus frequency. Normalised DPOAE amplitude values <0 dB were responses where the amplitude was below the control mean amplitude; values >0 dB were responses where the amplitude was higher than the control mean amplitude.

*Response amplitude slopes*. ABR W1 amplitude slope was measured from the 5–45 dB range above threshold and was expressed as µV / dB. Normalised ABR amplitude slope was calculated as each slope measurement divided by the mean slope from the control responses for each stimulus frequency. DPOAE amplitude slopes was measured from the 5 – 25 dB range above threshold and was expressed as dB / dB. Normalised DPOAE amplitude slope was calculated as each slope measurement divided by the mean slope from the control responses for each f2 stimulus frequency. Normalised slope values <1 were responses where the slope was lower than the control mean slope; values >1 were responses where the slope was higher than the control mean slope.

*ABR latency*. ABR wave P1 Latency was taken from the first positive ABR peak recorded at the highest stimulus level presented (95 dB SPL). Normalised latency was calculated as the latency for each response divided by the mean latency for control responses for each stimulus frequency. Normalised latency values <1 represent those shorter that the control mean latency; values >1 represent those longer than the control mean latency.

*Clustering of response thresholds*. The mean of each set of normalised thresholds, and the corresponding standard deviation (SD), was used to define an elliptical boundary for that set of results, with the ellipse centred on the mean normalised ABR & DPOAE thresholds, and it’s semi-major and semi-minor axes corresponding to twice the SD for the normalised ABR threshold mean and normalised DPOAE threshold mean, respectively. The numbers of points falling within the ellipse boundaries were counted using the MATLAB *inpolygon* function (MAT-LAB R2021a, The MathWorks, Inc., Natick, Massachusetts, USA).

## 3 Results

Ten mutant mouse lines with known pathologies were used to investigate a scheme for predicting the underlying pathology of hearing impairment in other mutant mouse lines.

### 3.1 ABR & DPOAE thresholds in three exemplar mutant lines

**Fig. 1:**
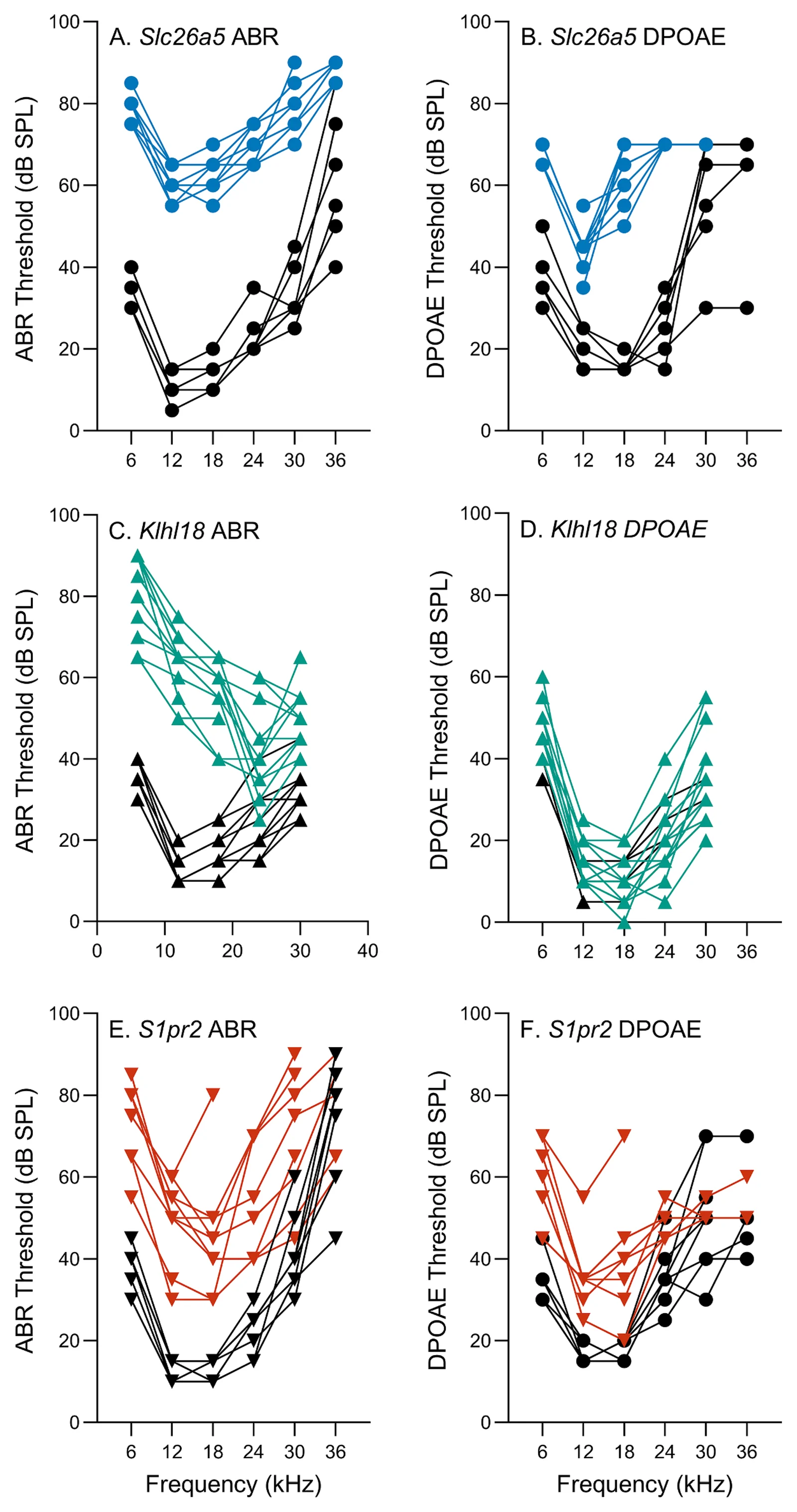
ABR & DPOAE Thresholds. Thresholds from ABRs are plotted in the left column (A,C,E); thresholds from DPOAEs plotted in the right column (B,D,F). Thresholds were obtained from *Slc26a5^tm1^* littermate control mice (*n* = 7 wildtype; A & B, black circles) and homozygous mutants (*n* = 11; A & B, blue circles) aged 4 weeks, *Klhl18^lowf^* littermate control mice (*n* = 3 wildtype, *n* = 9 heterozygote; C & D, black up-triangles) and homozygous mutants (*n* = 13; C & D, green up-triangles) aged 6 weeks, *S1pr2^stdf^* littermate controls (*n* = 1 wildtype, *n* = 8 heterozygote; E & F, black down-triangles) and homozygous mutants (*n* = 9; E & F, red down-triangles) aged 4 weeks.

Fig. 1 plots ABR and DPOAE thresholds obtained across all frequencies tested for the *Slc26a5^tm1^* (prestin), *Klhl18^lowf^* and *S1pr2^stdf^* homozygous mutant mice and their littermate controls. ABR thresholds obtained from these mutant mice show the same patterns of elevation (hearing impairment) to those published previously for these mutants (Lachgar-Ruiz et al. 2024, Ingham et al., 2021, Ingham et al. 2016, respectively). Thresholds are plotted from only those stimuli that evoked responses below the upper limits of the sound delivery systems.

At 4 weeks old, *Slc26a5^tm1^* mutant mice showed an increase in thresholds which was present across all frequencies tested and was relatively consistent between individual animals, for both ABR (Fig. 1A) and DPOAE recordings (Fig. 1B). *Klhl18^lowf^* mutant mice, aged 6 weeks, demonstrated loss of ABR thresholds which predominantly affected the lower frequencies tested (Fig. 1C), whereas DPOAE thresholds were comparable to littermate controls (Fig. 1D). At 4 weeks old, both ABR thresholds (Fig. 1E) and DPOAE thresholds (Fig. 1F) from *S1pr2^stdf^* mutant mice showed variable levels of elevation compared to thresholds seen in littermate control mice.

### 3.2 Normalised ABR & DPOAE thresholds in the three exemplar mutant lines

**Fig. 2:**
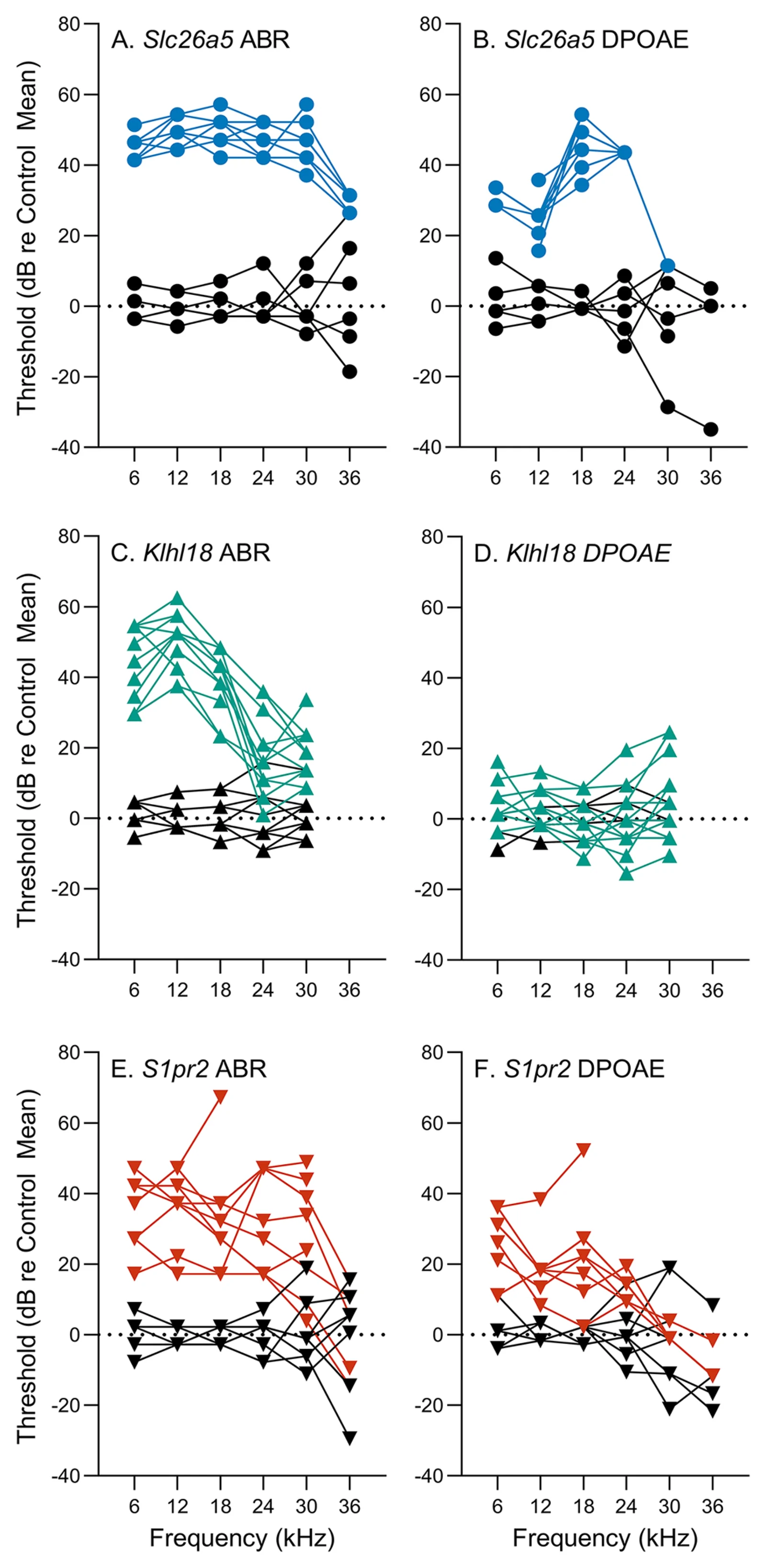
Normalised ABR & DPOAE Thresholds. Normalised thresholds from ABRs are plotted in the left column (A,C,E) and those from DPOAEs plotted in the right column (B,D,F). Numbers of mice from *Slc26a5^tm1^*, *Klhl18^lowf^* and *S1pr2^stdf^* colonies are the same as for Fig. 1.

Following the method used by Mills (2006), mutant and littermate control thresholds for each frequency, for ABRs and DPOAEs (separately) were normalised to the control mean threshold for that frequency. This simple procedure involved subtracting the control mean threshold for each frequency away from each individual threshold estimate for control and mutant mice. This produced normalised threshold data that clustered around 0 dB for each frequency for control responses whereas normalised thresholds for mutant responses reflect the degree of threshold elevation above the control mean (Fig. 2).

### 3.3 Comparison of normalised ABR & DPOAE thresholds

In Fig. 3, normalised DPOAE thresholds are plotted against normalised ABR thresholds, in the same way that Mills (2006) used to develop a scheme to diagnose hearing impairment pathology induced experimentally in the gerbil. The zones that Mills outlined to describe Acoustic/OHC, strial/EP and Neural/IHC pathologies are indicated in grey in the background on each plot of Fig. 3.

The normalised thresholds obtained from control mice are plotted in black and can be seen to form a roughly circular cluster centred on the ABR and DPOAE axes origins (0 dB). Whilst some responses from the mutant mice fall into the predicted location for the known mouse pathology, many responses from mutant mice do not fall into the expected zone on these plots. Proportions of responses falling in the various pathology zones for each mutant mouse line are given in Table 2.

### 3.4 Elliptical boundaries to cluster mutant thresholds

**Fig. 4:**
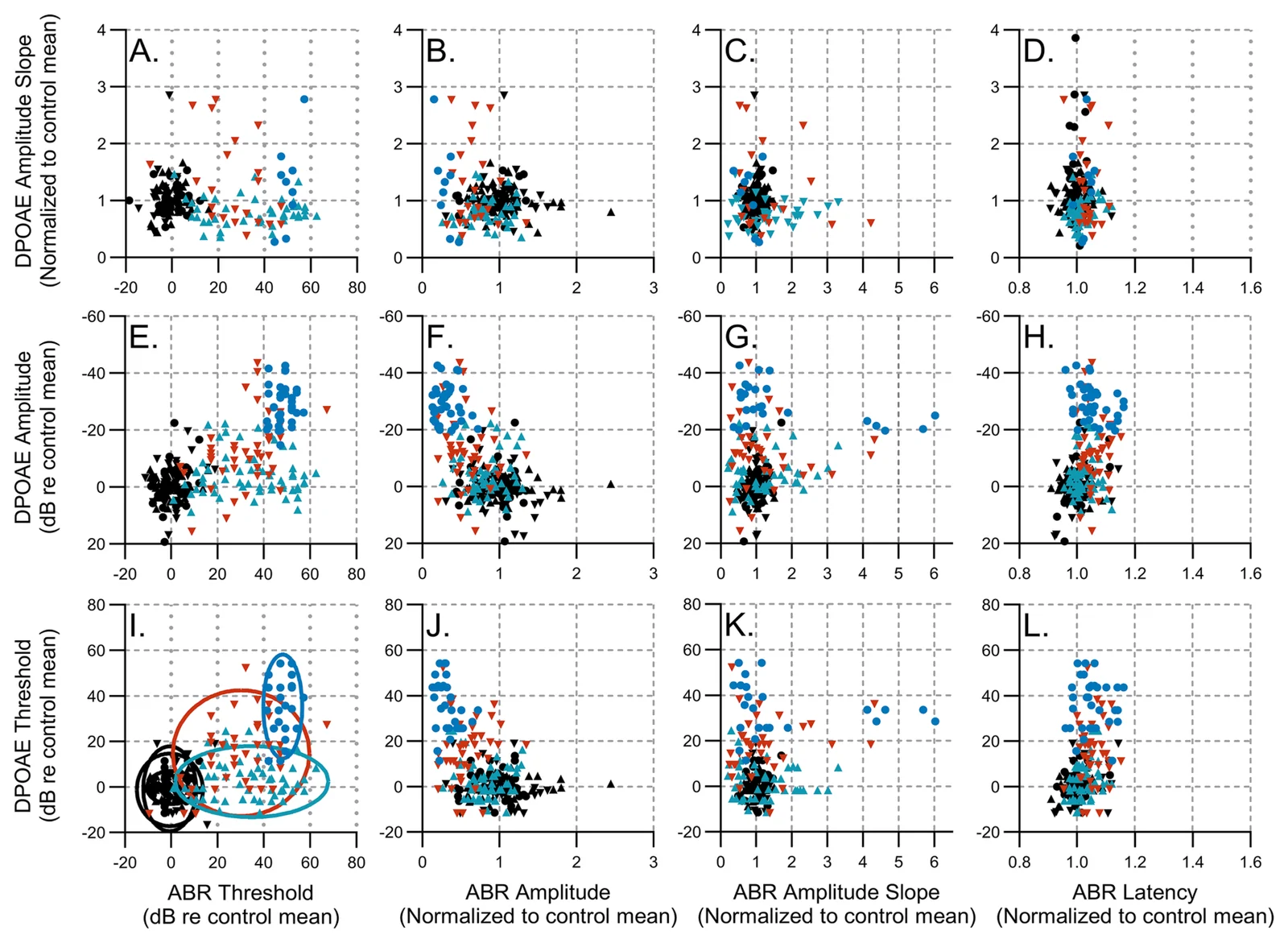
Comparisons of normalised ABR and DPOAE parameter measurements. Parameters were measured from the ABR and DPOAE responses recorded from the different mutant mouse lines, as described in the methods. (A-L). Normalised DPOAE parameters (threshold, amplitude at 75 dB f1 stimulus level, and amplitude slope) were plotted against normalised ABR parameters (threshold, wave 1 amplitude at 95 dB stimulus level, wave 1 amplitude slope and wave P1 latency at 95 dB stimulus level). In each plot responses from normal hearing littermate control mice are plotted as black symbols, with the symbol corresponding that used for the different mutant mice. Responses from *Slc26a5^tm1^* mice are plotted as circles, with the mutants plotted in blue. Responses from *Klhl18^lowf^* mice are plotted as up-triangles, with the mutants plotted in green. Responses from *S1pr2^stdf^* mice are plotted as down-triangles, with the mutants plotted in red. I. The ellipses plotted enclose the different control and mutant groups of responses, black indicating controls, blue indicating the *Slc26a5^tm1^* mutant mice, green indicating the *Klhl18^lowf^* mutant mice and red indicating the *S1pr2^stdf^* mutant mice (See also Supplementary Material Figure S1.).

Inspection of the mutant response distributions in plots of normalised DPOAE thresholds against normalised ABR threshold (Fig. 3) shows that these responses tend to fall into clusters rather than within the more linear zones predicted by Mills (2006). The distributions of these thresholds were used to drawn elliptical boundaries around the datasets from the mutant mouse lines, as described in the methods and are plotted in Fig. 4I. However, these boundaries show considerable over-lap, thereby reducing their effectiveness as a means of differentiating the different pathologies. The percentages of responses from each of the three pathologies in each of the three elliptical clusters are given in Table 2, showing that more points for each mutant fall into the ellipses than into the linear zones of Mills (2006). For example, of responses from the *S1pr2^stdf^* mutant mice (strial / metabolic pathology), 10 % fall into the ellipse for the control group, 51 % fall into the neural pathology ellipse, 12 % into the sensory / outer hair cell boundary and 5 % fall outside of all the ellipses. Thus, the ellipse for the strial/metabolic pathology shows reasonable sensitivity (proportion of all true positives falling within the ellipse) but very poor specificity due to the extensive overlap with other ellipses. In contrast, if the strial/metabolic ellipse is ignored, the ellipses for the other three groups (normal-hearing controls, IHC/neural, and OHC/sensory) show good sensitivity and specificity illustrated by the minimal overlap between their ellipses.

In addition to response threshold, we calculated the maximum amplitude of ABR wave 1 and the amplitude of the DPOAE at maximum stimulus level, the slope of ABR and DPOAE amplitude growth with increasing stimulus intensity, and the latency of the first positive peak in the ABR waveform. Comparison of these parameters with normalised ABR or DPOAE thresholds (Fig. 4A - L) did not show any clear differential between the inner ear pathologies of the mice tested here.

### 3.5 Development of hearing impairment

From previous studies on the *S1pr2^stdf^* and *Klhl18^lowf^* mutant lines (Ingham et al. 2016, 2021), we know that homozygous mutant mice from either line develop normal ABR thresholds after the onset of hearing at post-natal day (P)12, maintain these good thresholds until 3 weeks of age (P21) and then thresholds rise progressively over the following weeks, so that *S1pr2^stdf^* mutants show a variable degree of ABR threshold elevation across 6–36 kHz by just a week later, and *Klhl18^lowf^* mutants showed pronounced low frequency (<24 kHz) ABR threshold elevations by 6 weeks old.

**Fig. 5:**
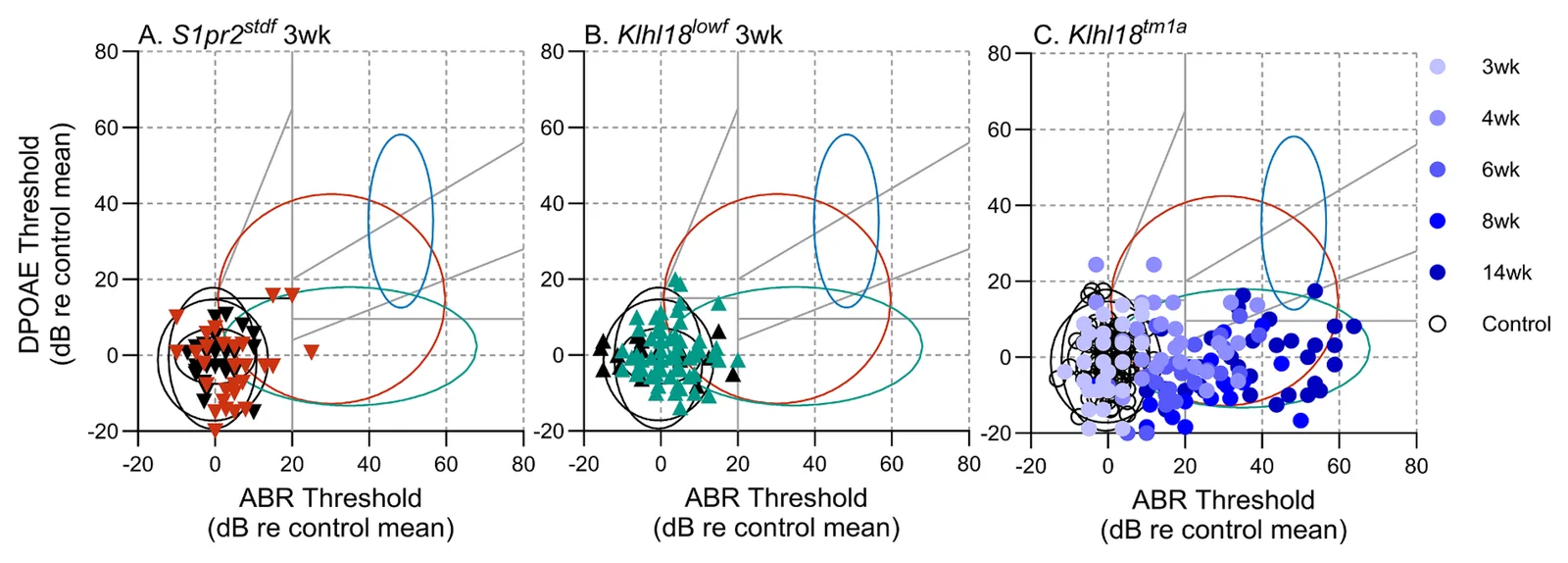
Clustering of ABR & DPOAE normalised thresholds during development. A. Results from 3 weeks old *S1pr2^stdf^* littermate control mice (*n* = 6 wildtypes; black circles) and homozygous mutants (*n* = 4; orange down-triangles). B. Results obtained from 3 weeks old *Klhl18^lowf^* littermate control mice (*n* = 8 heterozygotes; black circles) and homozygous mutants (*n* = 19; green up-triangles). C. Results obtained from a series of increasing age of *Klhl18^tm1a^* mice, aged 3 weeks (*n* = 4 wildtypes, *n* = 7 homozygous mutants), 4 weeks (*n* = 3 wildtypes, *n* = 5 heterozygotes, *n* = 8 homozygous mutants), 6 weeks (*n* = 6 wildtypes, *n* = 6 homozygous mutants), 8 weeks (*n* = 1 wildtypes, *n* = 5 heterozygotes, *n* = 6 homozygous mutants) and 14 weeks (*n* = 3 wildtypes, *n* = 5 heterozygotes, *n* = 7 homozygous mutants). Here, all littermate control mice (wildtypes and heterozygotes) are indicated by black circles and homozygous mutants indicated by increasingly dark purple circles, as indicated on the legend.

Fig. 5A & B illustrates normalised ABR thresholds and normalised DPOAE thresholds from mutants and littermate control mice from the *S1pr2^stdf^* & *Klhl18^lowf^* lines, respectively, recorded at P21. The normal hearing thresholds in these mutant mice at this age is reflected in the clustering of the mutant responses around the origin on both axes. In both cases, very few responses fall outside the normal hearing clusters (copied from Fig. 4I), and the distribution stands in contrast to the rightward shift in normalised ABR thresholds seen in these mutant mice at older ages. This pattern is also seen in Fig. 5C, showing responses from an increasing age series of recordings from mice carrying of a targeted mutant allele of *Klhl18, Klhl18^tm1a^*. These mice exhibit a primary IHC / neural pathology, and a progressive rightward shift in responses is shown as their hearing impairment progresses from 3 weeks to 14 weeks old.

### 3.6 Other mutant lines exhibiting hearing-impairment

**Fig. 6:**
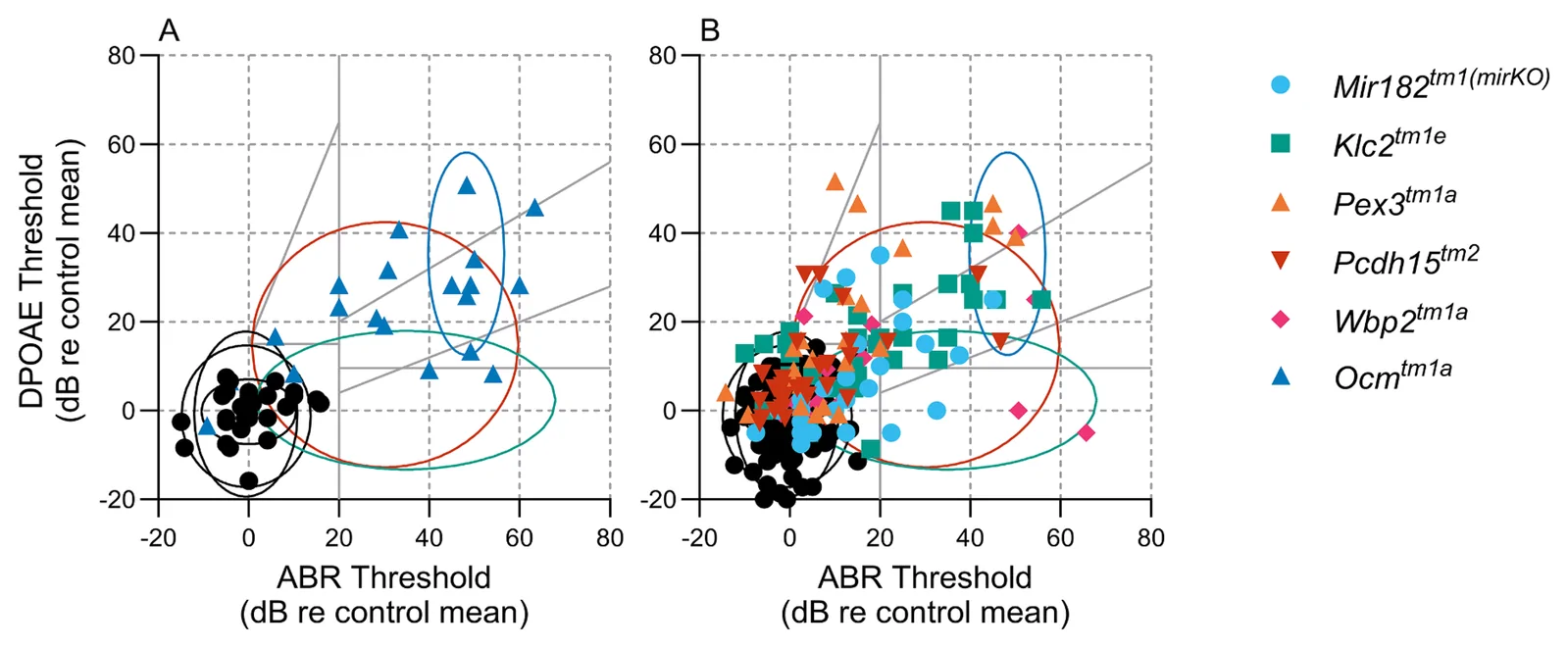
Distribution of ABR & DPOAE normalised thresholds for other mouse mutants with hearing impairment. Normalised DPOAE thresholds are plotted against normalised ABR thresholds. The Mills-zone boundaries are indicated by grey lines, as in Fig. 3. The elliptical cluster boundaries from the 3 exemplar pathology lines are indicated by the same-coloured lines as in Fig. 4I. A. Results from *Ocm^tm1e^* littermate control mice (*n* = 5 wildtypes, *n* = 1 heterozygote; black circles) and homozygous mutants with a sensory defect (*n* = 5; blue circles). B. Results from five mutant lines with mixed sensory/neural defects. *Klc2^tm1e^* littermate control mice (*n* = 7 wildtypes; black circles) and homozygous mutants (*n* = 8; green squares). *Mir182^ko^* littermate control mice (*n* = 2 wildtypes; black circles) and homozygous mutants (*n* = 7; blue circles). *Pex3^tm1a^* littermate control mice (*n* = 6 wildtypes; black circles) and homozygous mutants (*n* = 7; orange up-triangles). *Pcdh15^em2Wtsi^* littermate control mice (*n* = 9 wildtypes; black circles) and homozygous mutants (*n* = 8; red down-triangles). *Wbp2^tm2a^* littermate control mice (*n* = 4 wildtypes, *n* = 4 heterozygotes; black circles) and homozygous mutants (*n* = 6; red diamonds).

To further evaluate the Mills scheme and the elliptical cluster scheme, we have tested mice carrying a range of other mutant alleles resulting in hearing impairment. These results are summarised in Fig. 6 and Table 2.

*Ocm^tm1e^* mutants generally have very high threshold ABR responses at frequencies above 12 kHz, or no response at all (Lachgar-Ruiz et al. 2024). Low frequency ABR thresholds are moderately elevated too. *Ocm* is highly expressed in OHCs, suggesting a sensory pathology. Whilst 20 – 25 % of responses fall into the OHC / sensory regions of the two schemes, most responses fall elsewhere, with 45 – 80 % of responses falling into the strial / metabolic regions of the Mills-zone and ellipse clustering schemes, respectively (Fig. 6A, Table 2).

*Klc2^tm1e^* mutant mice show a predominantly low frequency hearing impairment, involving OHC dysfunction and loss as well as strong expression in the spiral ganglion and signs of abnormal Kcnma1 expression in IHCs indicating a sensory/neural pathology (Ingham et al. 2019). Neither scheme accurately reflect this, with only 10 % and 15 % of responses falling into the sensory Mills-zone or elliptical cluster and 0 % and 29 % falling into the neural Mills-zone or elliptical cluster, respectively (Fig. 6A, Table 2).

*Mir182* is part of the *Mir96/183/182* cluster of microRNA-encoding genes expressed together in all sensory hair cells, implying a sensory/ neural function. *Mir182^ko^* mutant mice exhibit mild high frequency elevations of ABR threshold (Lewis et al., 2020) and 60 % of responses fall into the normal hearing Mills-zone. However, 43 % and 54 % of responses fall in the neural pathology ellipse and strial pathology ellipse, respectively (Fig. 6A, Table 2).

The three remaining mutant mouse lines (*Pex3^tm1a^*, *Pcdh15^em2Wtsi^*, *Wbp2^tm2a^*) all show high frequency ABR threshold elevations, with relatively normal thresholds at 12 kHz and below, and all with evidence of both sensory and neural components to their pathology (Kochaj et al. 2022; Buniello et al., 2016; and unpublished data). Most of the responses from these lines fall into the normal hearing zone, and a large proportion fall into the normal hearing elliptical clusters. However, many also fall into the strial / metabolic hearing elliptical cluster (Fig. 6A, Table 2).

Rows 1–5 indicated results from the 3 exemplar mutant lines. Rows 6–11 indicate results from the 6 further mutant lines tested. Number of responses refers to the number of individual frequency/mouse points included. Pre-Clin refers to a zone defined as Pre-clinical acoustic damage by Mills (2006) as indicated in Fig. 3.

## 4 Discussion

Here, we present our analysis of non-invasive physiological tests in mutant mice with known underlying sites-of-lesion aimed at testing a scheme proposed to differentially diagnose a strial dysfunction / metabolic pathology from other cochlear pathologies. We found that the mouse mutants tested with a primary strial dysfunction (*S1pr2^stdf^*) did not replicate the findings in gerbils with a furosemide-induced reduction in EP previously published (Mills 2006). Datapoints from mice with a prestin mutation (*Slc26a5^tm1^*) affecting outer hair cells expected to lead to a sensory pathology also were more widely spread than predicted by the published gerbil results.

The new data from three exemplar mouse mutants representing the three main classes of cochlear pathology were next used to define ellipses that contained the majority of the datapoints of each type. These ellipses gave a good diagnostic differentiation between OHC/sensory and IHC/neural hearing impairments. However, there was extensive overlap between the stria/metabolic ellipse and ellipses for hearing impairment from the OHC/sensory or IHC/neural pathologies. Therefore, the ellipses were unable to predict accurately the individual mice with a strial dysfunction. The *S1pr2^stdf^* mutants gave a wide range of ABR and DPOAE thresholds spread around the plot (Fig. 3C, 4), suggesting that the relationship between different aspects of the hearing loss varied between individuals. The reason for this is unclear, as the mutants were all on a uniform inbred genetic background and were maintained in the same environment, often as cagemates. It would be useful to test the hypothesis put forward by Mills (2006) using other mouse mutant lines with strial dysfunction as the primary pathology.

Examination of DPOAE and ABR thresholds in several other mutant lines with sensory or sensory/neural sites-of-lesion revealed a wide scatter of datapoints in all six mutants. Again, the reason for this scatter is not obvious. Different points reflect different frequencies so it would not be surprising if there was some scatter as frequencies show different absolute thresholds even in normal-hearing mice, but the poor correlation between DPOAE and ABR thresholds was not expected.

There could be a number of reasons that the results of Mills (2006) are different from the current study. The exact measurement techniques for ABR and DPOAE are different, but any response differences resulting from this would be accounted for during the threshold normalisation step. Mills (2006) used noise-exposure or chronic furosemide administration to induce hearing impairment in gerbils. It is known that such experimental procedures may not induce a specific, single site-of-lesion; for example, noise exposure can affect multiple aspects of normal cochlear function, such as stereocilia damage to hair cells (Pickles et al. 1987), synaptopathic changes (Kujawa and Liberman 2009), or changes to the stria vascularis (Ohlemiller and Gagnon 2007). Species differences could also account for the differences in results patterns seen. Here, we use mice with known pathological lesions resulting from specific mutations. For the *Klhl18^lowf^* and *S1pr2^stdf^* mutant lines, these pathologies induce an age-dependent progressive hearing loss, with the degree of threshold elevation worsening with increasing age of the mice. We have chosen test ages (4 weeks old for *Slc26a5^tm1^* mutants, 6 weeks old for *Klhl18^lowf^* mutants and 4 weeks old for *S1pr2^stdf^* mutants) where previously published data (Ingham et al. 2016, 2021; Lachgar-Ruiz et al. 2024) suggests a single site-of-lesion (OHC dysfunction, IHC dysfunction, and reduced EP, respectively) for these mutant lines with no obvious secondary pathology such as hair cell degeneration. However, it may be that we are measuring from mice with a partially mixed pathology which affects cochlear function in a more complex manner and alters the resulting physiological responses.

We have used a mutant with a primary IHC defect (*Klhl18^lowf^*) as an example of an IHC/neural defect in this study, because current methods used in clinical settings do not distinguish between an IHC defect, a synaptic defect, or a neural defect such as a myelination problem. These are often grouped together as cases of auditory neuropathy. Further work will be needed to explore methods for distinguishing between these different types of pathology.

The clustering scheme presented here does not provide a conclusive method to distinguish between strial/metabolic and other types of hearing impairment. Further work is needed to design tests that may be helpful for translation into a clinical environment. Such tests will need to use non-invasive, and preferably objective, methods such as auditory evoked potentials or otoacoustics emissions for clinical translation to differentiate inner ear sites of lesion.

## Supplementary Material

Supplementary material associated with this article can be found, in the online version, at doi:10.1016/j.heares.2025.109391.

Supplementary data

## Figures and Tables

**Table 1:** Summary of hearing-impaired mutant mouse lines used in this study.

Gene / Allele (shortened name)	Age (weeks)	Cochlear Site of Lesion	Pathology	Reference
***S1pr2^stdf^***	3, 4	Stria Vascularis.	Metabolic	Ingham et al.2016
***Klhl18^lowf^***	3, 6	Inner Hair Cell, abnormal apical stereocilia	Neural	Ingham et al., 2021
***Klhl18^tm1a(KOMP)Wtsi^*** (*Klhl18^tm1a^*)	3, 4, 6, 8, 14	Inner Hair Cell, abnormal apical stereocilia	Neural	Ingham et al., 2021
***Slc26a5^tm1(EGFP/Cre/ERT2)Wtsi^*** (*Slc26a5^tm1^*)	4	Outer Hair Cells.	Sensory	Lachgar-Ruiz et al. 2024
***Ocm^tm1e(EUCOMM)Wtsi^*** (*Ocm^tm1e^*)	8	Outer Hair Cells	Sensory	Lachgar-Ruiz et al. 2024
***Klc2^tm1e(EUCOMM)Wtsi^*** (*Klc2^tm1e^*)	3	Outer Hair Cells. Spiral ganglion	Sensory / Neural	Ingham et al.2019
***Mir182^tm1Hmpr/Wtsi^*** (*Mir182^ko^*)	8	Inner & Outer Hair Cells	Sensory / Neural	Lewis et al., 2020
***Pex3^tm1a (EUCOMM)Wtsi^*** (*Pex3^tm1a^*)	4	Inner & Outer Hair Cells, Reduced synapse counts	Sensory / Neural	Kochaj et al.2022
***Pcdh15^em2Wtsi^*** *(Pcdh15^em2^)*	8	Inner & Outer Hair Cells.	Sensory / Neural	(unpublished)
***Wbp2^tm2a(EUCOMM)Wtsi^*** (*Wbp2^tm2a^*)	4	Abnormal synapses & innervation	Sensory / Neural	Buniello et al., 2016

**Table 2:** Proportions of responses in Mills-zones & elliptical cluster boundaries.

Gene / Allele	Age (weeks)	Number of Responses	% Responses in Mills-zones					% Responses in Elliptical Cluster Boundaries				
*Exemplar Mutants*			Normal	Pre-Clin	OHC	Strial	Neural	Normal	Neural	Strial	OHC	None
***Klhl18^lowf^***	6	64	21.9	1.6	1.6	9.4	64.1	4.7	89.1	89.1	0.0	0.0
***Slc26a5^tm1^***	4	38	0.0	0.0	31.6	63.2	0.0	0.0	7.9	50.0	92.1	2.6
***S1pr2^stdf^***	4	41	22.0	9.8	7.3	43.9	17.1	9.8	51.2	95.1	12.2	4.9
*Normal Hearing Mutants*												
***Klhl18^lowf^***	3	95	97.9	1.1	0.0	0.0	1.1	54.7	38.9	28.4	0.0	1.1
***S1pr2^stdf^***	3	31	87.1	6.5	0.0	3.2	3.2	38.7	32.3	29.0	0.0	0.0
*Other Hearing Loss Mutants*												
***Klc2^tm1e^***	3	41	39.0	14.6	9.8	29.3	0.0	4.9	29.3	63.4	14.6	2.4
***Mir182^ko^***	8	35	60.0	11.4	8.6	14.3	5.7	37.1	42.9	54.3	2.9	0.0
***Ocm^tm1e^***	8	20	15.0	5.0	20.0	45.0	10.0	5.0	25.0	80.0	25.0	10.0
***Pex3^tm1a^***	4	24	54.2	20.8	12.5	8.3	0.0	25.0	25.0	50.0	12.5	16.7
***Pcdh15^em2Wtsi^***	8	39	79.5	10.3	0.0	7.7	0.0	56.4	25.6	38.5	5.1	2.6
***Wbp2^tm2a^***	4	24	70.8	12.5	0.0	8.3	8.3	33.3	54.2	62.5	8.3	0.0

## Data Availability

All mouse mutants used are available from public archives. Auditory data are available from the authors on request.

## References

[R1] Buniello A, Ingham NJ, Lewis MA, Huma AC, Martinez-Vega R, Varela-Nieto I, Vizcay-Barrena G, Fleck RA, Houston O, Bardhan T, Johnson SL (2016). Wbp2 is required for normal glutamatergic synapses in the cochlea and is crucial for hearing. EMBO Mol Med.

[R2] Ingham NJ, Banafshe N, Panganiban C, Crunden JL, Chen J, Lewis MA, Steel KP (2021). Inner hair cell dysfunction in *Klhl18* mutant mice leads to low frequency progressive hearing loss. PLoS One.

[R3] Ingham NJ, Pearson S, Steel KP (2011). Using the auditory brainstem response (ABR) to determine sensitivity of hearing in mutant mice. Curr Protoc Mouse Biol.

[R4] Ingham NJ, Carlisle F, Pearson S, Lewis MA, Buniello A, Chen J, Isaacson RL, Pass J, White JK, Dawson SJ, Steel KP (2016). *S1PR2* variants associated with auditory function in humans and endocochlear potential decline in mouse. Sci Rep.

[R5] Ingham NJ, Pearson SA, Vancollie VE, Rook V, Lewis MA, Chen J, Buniello A, Martelletti E, Preite L, Lam CC, Weiss FD (2019). Mouse screen reveals multiple new genes underlying mouse and human hearing loss. PLoS Biol.

[R6] Kochaj RM, Martelletti E, Ingham NJ, Buniello A, Sousa BC, Wakelam MJO, Lopez-Clavijo AF, Steel KP (2022). The effect of a *Pex3* mutation on hearing and lipid content of the inner ear. Cells.

[R7] Kujawa SG, Liberman MC (2009). Adding insult to injury: cochlear nerve degeneration after “temporary” noise-induced hearing loss. J Neurosci.

[R8] Lachgar-Ruiz M, Ingham NJ, Martelletti E, Chen J, James E, Panganiban CH, Lewis MA, Steel KP (2024). Two new mouse alleles of Ocm and Slc26a5. Hear Res.

[R9] Lewis MA, Di Domenico F, Ingham NJ, Prosser HM, Steel KP (2020). Hearing impairment due to *Mir183/96/182* mutations suggests both loss and gain of function effects. Dis Model Mech.

[R10] Lewis MA, Ingham NJ, Chen J, Pearson S, Di Domenico F, Rekhi S, Allen R, Drake M, Willaert A, Rook V, Pass J (2022). Identification and characterisation of spontaneous mutations causing deafness from a targeted knockout programme. BMC Biol.

[R11] Lv J, Wang H, Cheng X, Chen Y, Wang D, Zhang L, Cao Q, Tang H, Hu S, Gao K, Xun M (2024). AAV1-hOTOF gene therapy for autosomal recessive deafness 9: a single-arm trial. Lancet.

[R12] Mills DM (2006). Determining the cause of hearing loss: differential diagnosis using a comparison of audiometric and otoacoustic emission responses. Ear Hear.

[R13] Mills DM, Schmiedt RA (2004). Metabolic presbycusis: differential changes in auditory brainstem and otoacoustic emission responses with chronic furosemide application in the gerbil. J Assoc Res Otolaryngol.

[R14] Ohlemiller KK, Gagnon PM (2007). Genetic dependence of cochlear cells and structures injured by noise. Hear Res.

[R15] Pickles JO, Osbourne MP, Comis SD (1987). Vulnerability of tip links between stereocilia to acoustic trauma in the guinea pig. Hear Res.

[R16] Qi J, Tan F, Zhang L, Lu L, Zhang S, Zhai Y, Lu Y, Qian X, Dong W, Zhou Y, Zhang Z (2024). AAV-mediated gene therapy restores hearing in patients with DFNB9 deafness. Adv Sci.

[R17] Schuknecht HF, Gacek MR (1993). Cochlear pathology in presbycusis. Ann Otol Rhinol Laryngol.

[R18] Schuknecht HF (1994). Auditory and cytocochlear correlates of inner ear disorders. Otolaryngol Head Neck Surg.

[R19] Vaden KI, Eckert MA, Matthews LJ, Schmiedt RA, Dubno JR (2022). Metabolic and sensory components of age-related hearing loss. J Assoc Res Otolaryngol.

[R20] Wang H, Chen Y, Lv J, Cheng X, Cao Q, Wang D, Zhang L, Zhu B, Shen M, Xu C, Xun M (2024). Bilateral gene therapy in children with autosomal recessive deafness 9: single-arm trial results. Nat Med.

[R21] White JK, Gerdin AK, Karp NA, Ryder E, Buljan M, Bussell JN, Salisbury J, Clare S, Ingham NJ, Podrini C, Houghton R (2013). Genome-wide generation and systematic phenotyping of knockout mice reveals new roles for many genes. Cell.

