# Supplementary material for: Non-invasive testing to diagnose the underlying auditory pathology of hearing impairment
Source: Hear Res. Author manuscript; Available in PMC 2026 Oct 19. (PMC7619599; doi:10.1016/j.heares.2025.109391)
Supplement: Supplementary file 1 [file EMS213875-supplement-Supplementary_data.pdf]

Supplementary Information.

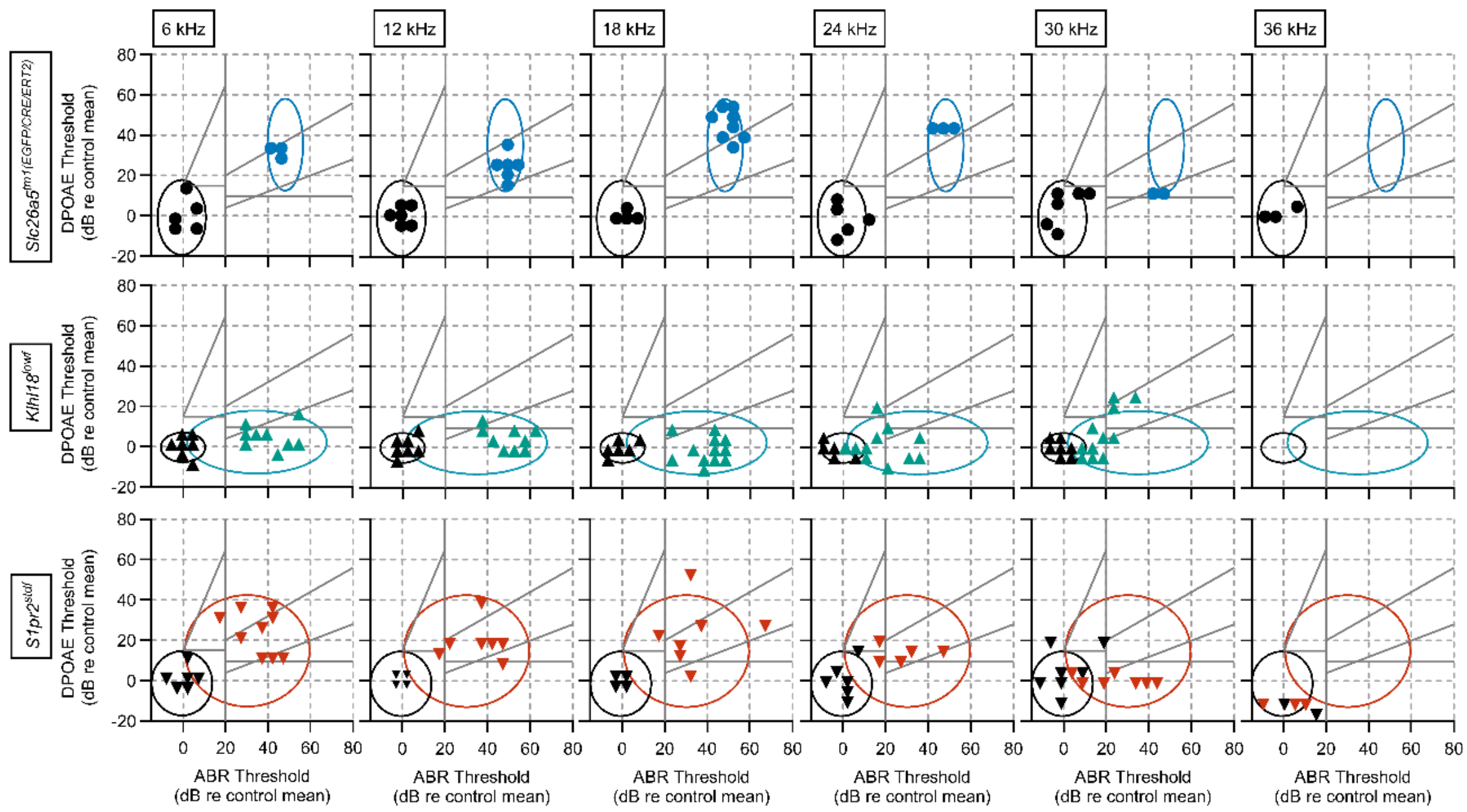

Figure S1. Normalised ABR and DPOAE thresholds, separated for stimulus frequency. The normalised ABR and DPOAE thresholds show in Figure 3 are replotted to show responses to stimuli of 6 kHz to 36 kHz in each column from left to right across the figure. As in Figure 3, responses from control animals are plotted in black with responses from mutant mice plotted as coloured symbols. Mice from the *Slc26a5^{tm1}* line are plotted as blue circles, mice from the *Klhl18^{lowf}* line plotted as green up-triangles and mice from the *S1pr2^{stdf}* line plotted as red down-triangles. The grey lines indicate the zones for different pathologies proposed by Mills (2006). Ellipses are replotted from Figure 4I, to enclose the different control and mutant groups of responses across all stimulus frequencies, black indicating controls, blue indicating *Slc26a5^{tm1}* mutant mice, green indicating *Klhl18^{lowf}* mutant mice and red indicating *S1pr2^{stdf}* mutant mice.
